# Comparative Utility of Tear Film and Ocular Surface Parameters in Differentiating Sjögren's Disease Dry Eye From Non-Sjögren's Dry Eye

**DOI:** 10.1167/tvst.15.9.17

**Published:** 2026-09-25

**Authors:** Meron Haile, Michael X. Lin, Julia Zeng, Gavin Li, David Cui, Michelle Hessen, Michela Montecchi-Palmer, Esen K. Akpek

**Affiliations:** 1 The Wilmer Eye Institute, Johns Hopkins University School of Medicine, Baltimore, MD, USA; 2 University of Maryland School of Medicine, Baltimore, MD, USA; 3 Stritch School of Medicine, Loyola University, Chicago, IL, USA; 4 Massachusetts Eye and Ear Infirmary, Harvard Medical School, Boston, MA, USA; 5 The University of North Carolina, Chapel Hill, NC, USA; 6 Alcon Inc., Fort Worth, TX, USA; 7 The Foster Center for Ocular Immunology and Inflammation, Duke University School of Medicine, Durham, NC, USA

**Keywords:** Sjögren disease (SjD), dry eye disease, ocular surface staining, schirmer test, aging

## Abstract

**Purpose:**

Tear production and ocular surface damage are equally weighted in the 2016 classification criteria for Sjögren's disease (SjD). We compared Schirmer testing versus ocular surface staining to distinguish SjD from non-SjD dry eye.

**Methods:**

Patients with SjD dry eye (*n* = 20), non-SjD dry eye (*n* = 47), and controls (*n* = 29) were evaluated in this cross-sectional study. Schirmer testing assessed tear production; ocular surface staining was graded using the Sjögren’s International Collaborative Clinical Alliance (SICCA) and National Eye Institute (NEI) systems. Analyses included age-stratified comparisons, receiver operating characteristic (ROC) analyses, and multivariable regression.

**Results:**

Overall, mean Schirmer values did not differ between SjD and non-SjD dry eye but were lower in SjD among participants <60 years (8.78 ± 6.24 vs. 18.00 ± 10.04 mm, *P* = 0.018), not ≥60 years (15.27 ± 11.01 vs. 14.00 ± 8.28 mm, *P* = 0.699). Schirmer ≤5 mm threshold differentiated SjD only in the younger group (<60 years: 55.6% vs. 5.0%, *P* = 0.014; ≥60 years: 18.2% vs. 25.9%, *P* = 0.145), and Schirmer testing showed poor overall discrimination, particularly among older participants (area under the curve [AUC] = 0.602; <60 years 0.772; ≥60 years 0.453). By contrast, SICCA corneal staining demonstrated consistently stronger discrimination (overall AUC 0.803; <60 years 0.872; ≥60 years 0.754). In multivariable models, NEI conjunctival staining (odds ratio [OR] = 1.23, *P* = 0.008) and SICCA corneal staining (OR = 1.83, *P* = 0.046), but not Schirmer testing (OR = 1.01, *P* = 0.888), were associated with SjD.

**Conclusions:**

Ocular surface staining was more strongly associated with classified SjD status in this cohort, whereas Schirmer testing showed age-dependent performance.

**Translational Relevance:**

Ocular surface staining may be a more reliable marker of Sjögren's dry eye than Schirmer testing, with implications for clinical evaluation.

## Introduction

An estimated 10% of patients with clinically significant dry eye have underlying Sjögren's disease (SjD), yet only approximately half carry a formal diagnosis at the time of presentation to eye care clinics.^1^^–^^3^ Early recognition is clinically important, as SjD dry eye may require different therapeutic considerations and untreated systemic disease may lead to various extraglandular complications, including increased lymphoma risk.^1^^–^^3^ As the first point of contact for many of these patients, ophthalmologists are uniquely positioned to recognize clinical features suggestive of SjD and to initiate timely diagnostic evaluations.^4^^,^^5^

Currently, distinguishing SjD dry eye from non-SjD dry eye remains challenging due to the heterogeneity of disease manifestations and variability in the performance and interpretation of commonly used diagnostic tests.^6^^–^^8^ In real-world settings, dry eye diagnosis and management are primarily driven by patient-reported symptoms and the application of objective tests varies considerably among clinicians.^9^^,^^10^ Corneal fluorescein staining is widely utilized, whereas Schirmer testing and conjunctival lissamine green staining are less consistently performed due to a variety of reasons, including time constraints, workflow limitations, and suboptimal reimbursement for comprehensive dry eye evaluations.^4^^,^^8^^,^^11^

Importantly, discrepancies exist between major diagnostic frameworks. The Tear Film and Ocular Surface Society Dry Eye Workshop (TFOS DEWS) does not include Schirmer testing among its core recommended assessments, whereas the 2016 American College of Rheumatology/European League Against Rheumatism (ACR–EULAR) classification criteria for SjD assign equal weight to Schirmer testing and ocular surface staining scores.^12^^–^^14^ Prior studies have shown that individual diagnostic modalities demonstrate variable associations with SjD, and that no single clinical test consistently distinguishes SjD dry eye from non-SjD dry eye.^8^^,^^11^ Moreover, age-related changes in tear production and ocular surface integrity may significantly influence test performance, particularly in older individuals, further complicating interpretation and reducing diagnostic precision.^12^^,^^13^

As a result, uncertainty persists regarding which physician-measured ocular surface and tear film parameter(s) offer the greatest clinical utility for identifying SjD in real-world practice. Accordingly, the objective of this study was to compare the diagnostic performance of recommended ocular surface and tear film parameters in patients with dry eye disease, with the aim of identifying the most informative and reliable clinical signs for differentiating SjD dry eye from non-SjD dry eye.

## Participants and Methods

We conducted a cross-sectional analysis of a clinic-based sample of patients with dry eye evaluated at the Ocular Surface Disease Clinic of the Wilmer Eye Institute at the Johns Hopkins University School of Medicine. The study protocol was approved by the Johns Hopkins Institutional Review Board (IRB00247104). All participants provided written informed consent prior to enrollment. The study adhered to the tenets of the Declaration of Helsinki.

### Study Population and Group Classification

Adult participants (≥18 years) with a prior diagnosis of dry eye established by an eye care provider within 6 months of presentation were recruited. Participants were categorized into SjD dry eye and non-SjD dry eye groups based on clinical and laboratory criteria. SjD dry eye was defined according to the 2016 ACR-EULAR classification criteria, requiring either anti-SSA positivity or minor salivary gland biopsy with focal lymphocytic sialadenitis (focus score ≥1 focus per 4 mm²), in combination with at least one objective feature of dryness (ocular staining score ≥5, Schirmer ≤5 mm/5 minutes, or unstimulated salivary flow rate ≤0.1 mL/min).^14^

Participants classified as non-SjD dry eye had negative serologic testing as well as negative minor salivary gland biopsy results to definitively exclude patients with SjD with false negative serology. Control participants were recruited from accompanying individuals in the same clinical setting and were selected to approximate the age distribution of the dry eye cohort. However, complete age matching was not feasible because recruitment occurred within an ophthalmology clinic population, where the availability of older individuals without dry eye was limited due to the increasing prevalence of dry eye disease with age.^3^^,^^11^^,^^12^ Controls were required to have no history of dry eye disease, no autoimmune or inflammatory conditions, and were asymptomatic (Ocular Surface Disease Index score <13).

### Exclusion Criteria

Participants were excluded if they had conditions or exposures that could confound assessment of tear film or ocular surface parameters. These included contact lens use within 10 days prior to evaluation, intraocular surgery within 3 months, in-office eyelid procedures for dry eye within 30 days, or prior corneal or eyelid surgery within 12 months. Participants using medications known to affect tear production or ocular surface integrity (including systemic antihistamines, diuretics, or recent topical ocular medications other than artificial tears) were excluded. Artificial tear use within 2 hours prior to examination was also an exclusion criterion.

Additional exclusions included systemic or ocular conditions affecting the ocular surface (diabetes and neurologic disease), prior cosmetic chemodenervation, autoimmune or inflammatory conditions (other than SjD), infectious or cicatrizing ocular surface disease, and conditions affecting lacrimal gland function (sarcoidosis, amyloidosis, graft-versus-host disease, hepatitis C, and HIV).

### Clinical Evaluation

All participants underwent a standardized ocular surface examination performed by a masked observer in a consistent fixed sequence to minimize the impact of prior testing on subsequent measurements. Schirmer testing was performed by a masked certified ophthalmic technician who served as the study coordinator. Ocular surface staining was independently graded by two masked investigators, both of whom were blinded to participants’ SjD status. Testing was conducted under controlled conditions; first with Schirmer testing without anesthesia over 5 minutes (TearFlo; Keeler, Malvern, PA). Following Schirmer testing, a 5-minute recovery period with the eyes closed was allowed before ocular surface staining was performed. Ocular surface epithelial staining was then performed using fluorescein for the cornea (BioGlo, Farmington Hills, MI) and lissamine green for the conjunctiva (GreenGlo, Farmington Hills, MI).

Ocular surface staining was graded using two established scoring systems. The Sjögren's International Collaborative Clinical Alliance (SICCA) ocular staining score includes corneal staining (0–6), based on punctate epithelial erosions graded from 0 to 3 with an additional point assigned for central, confluent, or filamentary staining, and conjunctival staining (0–6), based on lissamine green uptake in the nasal and temporal bulbar conjunctiva (each graded 0–3).^15^ The total SICCA score (range = 0–12) represents the sum of corneal and conjunctival components.^15^

The National Eye Institute (NEI) grading system evaluates corneal staining across five regions (central, superior, inferior, nasal, and temporal cornea; each graded 0–3, total 0–15) and conjunctival staining across six regions (nasal and temporal bulbar conjunctiva, each subdivided into superior, central, and inferior regions; each graded 0–3, total 0–18).^16^

### Outcome Measures

The primary outcomes included Schirmer test values and corneal and conjunctival staining scores using SICCA and NEI scales according to SjD dry eye status. To evaluate the influence of age on these measures, analyses were stratified a priori by age (<60 vs. ≥60 years). Age 60 years was selected a priori as the stratification threshold based on prior literature demonstrating accelerated age-related decline in lacrimal gland function and tear production.^17^ In addition to continuous measures, threshold-based variables were defined to align with 2016 ACR-EULAR classification criteria^14^ with recommended cut off points (Schirmer ≤5 mm and OSS ≥5) and presented in the analytic tables.

### Statistical Analysis

For all analyses, dry eye parameters were derived from the eye with more severe findings to reflect clinically relevant disease burden. Continuous variables are presented as mean ± standard deviation or median (interquartile range), and categorical variables as counts and percentages. Group comparisons used *t*-tests or Wilcoxon rank-sum tests for continuous variables and chi-square or Fisher exact tests for categorical variables, as appropriate, to compare differences between study groups.

Age-stratified analyses were performed to compare tear film and ocular surface parameters between SjD dry eye and non-SjD dry eye groups and between all dry eye participants versus controls within each age category (<60 and ≥60 years). To evaluate whether age was associated with individual parameters, univariable linear regression models were constructed with each parameter (Schirmer, SICCA corneal staining, NEI corneal staining, SICCA conjunctival staining, and NEI conjunctival staining scores) as the dependent variable and age as a continuous independent variable. Interaction terms between age and SjD dry eye status were included to determine whether age-related associations differed according to SjD status.

To identify which clinical parameters were independently associated with SjD dry eye, logistic regression models were constructed with SjD dry eye status as the dependent variable. Univariable models evaluated each ocular surface parameter individually. Multivariable models were constructed separately for SICCA- and NEI-based tear film and ocular surface parameters and included age (continuous), Schirmer test values, corneal staining scores, and conjunctival staining scores simultaneously to determine which measures retained independent discriminatory value after adjustment for covariates.

Receiver operating characteristic (ROC) curve analyses were performed to quantify the diagnostic performance of Schirmer testing and ocular surface staining parameters for identifying SjD dry eye. Areas under the ROC curve (AUROCs) were calculated for two prespecified comparisons: (1) SjD dry eye versus non-SjD dry eye only, to assess discrimination among patients with dry eye symptoms, and (2) SjD dry eye versus the overall cohort (non-SjD dry eye plus controls), to assess discrimination across the full study population. ROC analyses were also stratified by age (<60 vs. ≥60 years). Higher AUC values indicate better discrimination.

All analyses were conducted using Stata version 18.5 (StataCorp LLC, College Station, TX), and statistical significance was defined as a 2-sided *P* value < 0.05.

## Results

### Participant Characteristics

Demographic and clinical characteristics are presented in Table 1. A total of 96 participants were included, comprising 20 with SjD dry eye, 47 with non-SjD dry eye, and 29 controls. Control participants were younger than participants with dry eye (51.3 ± 13.3 years vs. 57.2 ± 15.5 and 60.3 ± 9.4 years, *P* = 0.029), whereas age did not differ between SjD and non-SjD dry eye groups (*P* = 0.402). A higher proportion of participants with SjD dry eye were female subjects compared with non-SjD dry eye (95.0% vs. 72.3%, *P* = 0.037).

**Table 1.** Demographic and Clinical Characteristics of Participants in a Clinic-Based Sample of Patients With Dry Eye Versus Controls

Variables	SjD Dry Eye, *n* = 20	Non-SjD Dry Eye, *n* = 47	Controls, *n* = 29	SjD Dry Eye vs. Non-SjD Dry Eye, *P* Value	All Dry Eye vs. Controls, *P* Value
Age, y, mean ± SD	60.3 ± 9.4	57.2 ± 15.5	51.3 ± 13.3	0.402	**0.029**
Age group, *n* (%)					
<60	9 (45.0)	20 (42.6)	20 (69.0)	0.052	0.116
≥60	11(55.0)	27 (57.4)	9 (31.0)		
Sex, *n* (%)					
Female	19 (95.0)	34 (72.3)	18 (62.1)	**0.037**	0.081
Male	1 (5.0)	13 (27.7)	11 (37.9)		
Race, *n* (%)					
White	13 (65.0)	37 (78.7)	18 (62.1)	0.276	0.531
Black	4 (20.0)	6 (12.8)	8 (27.6)		
Asian/Pacific Islander	3 (15.0)	2 (4.3)	2 (6.9)		
Other	0 (0)	2 (4.3)	1 (3.4)		
Schirmer, mm/5 min					
Mean ± SD, mm	8.4 ± 7.1	10.0 ± 7.2	15.5 ± 8.7	0.187	**0.001**
Median (IQR)	6 (4–10.5)	8 (5–12)	15 (7–20)		
≤5 mm, *n* (%)	7 (35.0)	8 (17.0)	1 (3.5)	0.106	**0.022**
Corneal Staining Score SICCA					
Mean ± SD	3.3 ± 1.4	2.0 ± 1.1	0.8 ± 0.5	**<0.001**	**<0.001**
Median (IQR)	3.5 (2–4.3)	2 (1–3)	1 (1–1)		
NEI					
Mean ± SD	5.65 ± 3.39	3.68 ± 2.47	1.00 ± 0.80	**0.024**	**<0.001**
Median (IQR)	5 (3–8)	4 (2–5)	1 (0–1)		
Conjunctival Staining Score SICCA					
Mean ± SD	4.35 ± 1.66	2.55 ± 1.78	1.10 ± 1.11	**<0.001**	**<0.001**
Median (IQR)	5 (3.5–5.5)	2 (1–4)	1 (0–2)		
NEI					
Mean ± SD	12.1 ± 4.59	7.09 ± 4.88	2.62 ± 2.74	**<0.001**	**<0.001**
Median (IQR)	13 (9–15)	6 (3–10)	2 (0–4)		
Combined Corneal and Conjunctival Staining SICCA				
Mean ± SD	7.45 ± 2.56	4.40 ± 2.67	1.90 ± 1.40	**<0.001**	**<0.001**
Median (IQR)	8 (6–9.5)	4 (3–6)	2 (1–3)		
SICCA ≥5, *n* (%)	17 (85.0)	21 (44.7)	1 (3.5)	**0.002**	**<0.001**
NEI					
Mean ± SD	17.6 ± 6.5	10.6 ± 6.8	3.6 ± 3.0	**<0.001**	**<0.001**
Median (IQR)	19 (13–22.5)	9 (6–16)	3 (1–5)		

IQR, interquartile range; NEI, National Eye Institute; SD, standard deviation; SICCA, Sjögren's International Collaborative Clinical Alliance; SjD, Sjögren's disease.

Data are presented as mean ± SD or median (interquartile range [IQR]) for continuous variables and *n* (%) for categorical variables.

Ocular surface staining was assessed using the SICCA and NEI grading systems.

Worst-eye values were used for all ocular surface parameters.

The SjD Dry Eye vs. non-SjD Dry Eye *P* value reflects comparisons between Sjögren's and non-SjD dry eye groups.

The All Dry Eye vs. Control *P* value reflects comparisons between all dry eye participants (SjD Dry Eye and non-SjD Dry Eye combined) and controls.

Statistical significance was defined as *P* < 0.05. Statistically significant *P* values are in bold.

**Table 2.** Utility of Tear Production and Ocular Surface Parameters Stratified by Age to Distinguish Sjögren’s in a Clinic-Based Sample of Patients With Dry Eye Versus Controls

	SjD Dry Eye		Non-SjD Dry Eye		Control			
Variables	<60	≥60	<60	≥60	<60	≥60	SjD Dry Eye vs. Non-SjD Dry Eye, *P* Value	All Dry Eye vs. Control, *P* Value
Participants, *n* (%)	9 (45.0)	11 (55.0)	20 (42.6)	27 (57.4)	20 (69.0)	9 (31.0)		
Schirmer, mm/5 min								
Mean ± SD	8.78 ± 6.24	15.27 ± 11.01	18.00 ± 10.04	14.00 ± 8.28	24.39 ± 8.92	15.33 ± 6.67	**0.018** **(<60)**, 0.699 (≥60)	**0.002 (<60)**, 0.765 (≥60)
Median (IQR)	5 (4–14)	10 (7–24)	15.5 (10.5–22)	13 (9–16)	25 (18–32)	15 (12–20)		
≤5 mm in either eye, *n* (%)	5 (55.56)	2 (18.18)	1 (5.00)	7 (25.93)	0 (0.00)	1 (11.11)	**0.014 (<60)**, 0.145 (≥60)	**0.001 (<60)**, **0.002 (≥60)**
Corneal Staining Score SICCA								
Mean ± SD	3.55 ± 1.51	3.00 ± 1.22	2.07 ± 1.21	1.80 ± 2.07	0.78 ± 0.44	0.80 ± 0.52	**<0.001** **(<60)**, **0.017 (≥60)**	**<0.001 (<60)**, **0.025 (≥60)**
Median (IQR)	4 (2–5)	3 (2–4)	2 (1–3)	2 (1–2.3)	1 (1–1)	1 (0.8–1)		
NEI								
Mean ± SD	4.89 ± 2.57	6.27 ± 3.95	3.00 ± 2.03	4.19 ± 2.68	1.00 ± 0.86	1.00 ± 0.71	**0.042 (<60)**, 0.067 (≥60)	**<0.001 (<60)**, **0.001 (≥60)**
Median (IQR)	5 (3–7)	5 (3–10)	2.5 (1–4.5)	4 (2–6)	1 (0–1.5)	1 (1–1)		
Conjunctival Staining Score SICCA								
Mean ± SD	4.78 ± 1.92	4.00 ± 1.41	2.35 ± 1.63	2.70 ± 1.90	1.20 ± 1.20	0.89 ± 0.93	**<0.001 (<60)**, **0.049 (≥60)**	**<0.001 (<60)**, **0.001 (≥60)**
Median (IQR)	5 (5–6)	4 (3–5)	2 (1–3.5)	2 (1–4)	1 (0–2)	1 (0–2)		
NEI								
Mean ± SD	13.67 ± 5.07	10.82 ± 3.92	6.55 ± 3.90	7.48 ± 5.54	2.85 ± 2.96	2.11 ± 2.26	**<0.001 (<60)**, 0.078 (≥60)	**<0.001 (<60)**, **0.001 (≥60)**
Median (IQR)	15 (13–17)	12 (8–15)	6.5 (3.5–8)	6 (2–12)	2 (0–3.5)	2 (0–4)		
Combined Corneal and Conjunctival Staining SICCA								
Mean ± SD	7.56 ± 2.88	7.36 ± 2.42	4.05 ± 2.44	4.67 ± 2.84	2.00 ± 1.49	1.67 ± 1.22	**<0.001 (<60)**, **0.009 (≥60)**	**<0.001 (<60)**, **0.0005 (≥60)**
Median (IQR)	9 (6–9)	7 (6–10)	4 (2–5.5)	5 (3–6)	2 (1–3)	2 (1–3)		
SICCA ≥5, *n* (%)	8 (88.89)	9 (81.82)	7 (35.00)	14 (51.85)	1 (5.00)	0 (0.00)	**0.014 (<60)**, 0.145 (≥60)	**0.001 (<60)**, **0.002 (≥60)**
NEI								
Mean ± SD	18.11 ± 6.75	17.09 ± 6.61	9.35 ± 5.70	11.48 ± 7.55	3.80 ± 3.17	3.00 ± 2.65	**<0.001 (<60), 0.039 (≥60)**	**<0.001 (<60), <0.001 (≥60)**
Median (IQR)	20 (15–22)	18 (12–23)	8.5 (5.5–13)	11 (6–17)	3 (1–5.5)	3 (1–5)		

Data are presented as mean ± SD or median (interquartile range [IQR]) for continuous variables and *n* (%) for categorical variables.

Ocular surface staining was assessed using the SICCA and NEI grading systems.

Worst-eye values were used for all ocular surface parameters.

Age groups were defined as <60 years and ≥60 years at the time of examination.

The SjD Dry Eye vs. non-SjD Dry Eye *P* value reflects comparisons between Sjögren's and non-Sjögren dry eye groups.

The All Dry Eye vs. Control *P* value reflects comparisons between all dry eye participants (SjD Dry Eye and non-SjD Dry Eye combined) and controls.

*P* values are shown as age-stratified results for patients <60 and ≥60 years, respectively.

Statistical significance was defined as *P* < 0.05. Statistically significant *P* values are in bold.

Compared with controls, participants with dry eye demonstrated worse tear production and higher ocular surface epithelial staining scores using both SICCA and NEI grading systems (all *P* < 0.001 for most comparisons). When comparing SjD dry eye and non-SjD dry eye groups, Schirmer test values did not differ significantly (8.4 ± 7.1 vs. 10.0 ± 7.2 mm, *P* = 0.187). In contrast, both SICCA and NEI-based staining measures were consistently higher in SjD dry eye compared with non-SjD dry eye, including SICCA corneal staining (*P* ≤ 0.001), NEI corneal staining (*P* = 0.024), and total conjunctival staining across both grading systems (both *P* ≤ 0.001). The proportion of participants meeting the SICCA-defined ocular staining score threshold ≥5 was significantly greater in SjD dry eye compared with non-SjD dry eye (85.0% vs. 44.7%, *P* = 0.002). Nasal and temporal conjunctival regions were evaluated separately and did not differ.

Notably, ocular surface staining was not uniformly absent among control participants as low levels of staining were also observed. Using the SICCA grading system, only 12 of 29 controls (41.4%) had no detectable staining (total OSS = 0 in either eye), whereas only 6 of 29 (20.7%) had no staining in either eye using the NEI system. No control participants demonstrated complete absence of staining in both eyes across either grading system. In both grading systems, a score of 0 allows presence of fewer than 10 punctate epithelial dots.^16^^,^^17^ Mean and median staining values in controls remained low but nonzero, including SICCA corneal staining (0.8 ± 0.5; median = 1 [0–2]), NEI corneal staining (1.0 ± 0.8; median = 1 [0–1]), SICCA conjunctival staining (1.1 ± 1.1; median = 1 [0–2]), and NEI conjunctival staining (2.62 ± 2.74; median = 2 [0–4]), indicating that mild staining was present even in asymptomatic individuals without a diagnosis of dry eye.

### Age-Stratified Analysis of Tear Production and Ocular Surface Parameters

Among participants <60 years, compared with controls, participants with dry eye demonstrated reduced tear production on Schirmer testing (18.68 ± 10.49 vs. 24.39 ± 8.92 mm, *P* = 0.002) and higher ocular surface staining scores across both SICCA and NEI grading systems (all *P* < 0.001; Table 2).

When comparing SjD and non-SjD dry eye in participants <60 years old, Schirmer values were lower in SjD dry eye (8.78 ± 6.24 vs. 18.00 ± 10.04 mm, *P* = 0.018), and more participants met the Schirmer ≤5 mm threshold (55.6% vs. 5.0%, *P* = 0.014). Ocular surface staining measures were likewise higher in SjD dry eye, including SICCA corneal staining (3.55 ± 1.51 vs. 2.07 ± 1.21, *P* < 0.001), NEI corneal staining (4.89 ± 2.57 vs. 3.00 ± 2.03, *P* = 0.042), SICCA conjunctival staining (4.78 ± 1.92 vs. 2.35 ± 1.63, *P* < 0.001), NEI conjunctival staining (13.67 ± 5.07 vs. 6.55 ± 3.90, *P* < 0.001), combined SICCA corneal and conjunctival staining (7.56 ± 2.88 vs. 4.05 ± 2.44, *P* < 0.001), and combined NEI corneal and conjunctival staining (18.11 ± 6.75 vs. 9.35 ± 5.70, *P* < 0.001). The SICCA-defined OSS ≥5 threshold was also more frequent in SjD dry eye (88.9% vs. 35.0%, *P* = 0.014).

Among participants ≥60 years of age, Schirmer values did not differ between participants with dry eye and controls (14.55 ± 8.56 vs. 15.33 ± 6.67 mm, *P* = 0.765). In contrast, ocular surface staining scores remained significantly higher in participants with dry eye compared with controls, across both grading systems, including corneal, conjunctival, and combined corneal and conjunctival staining measures (all *P* ≤ 0.025).

When comparing SjD and non-SjD dry eye in participants ≥60 years of age, Schirmer values and threshold performance (≤5 mm/5 minutes) did not differ. However, staining measures remained higher in SjD dry eye, particularly for SICCA corneal (*P* = 0.017), SICCA conjunctival (*P* = 0.049), SICCA combined corneal and conjunctival staining (*P* = 0.009), and NEI combined corneal and conjunctival staining (*P* = 0.039), whereas NEI corneal staining was not significantly different. In contrast to Schirmer threshold performance, the SICCA combined corneal and conjunctival staining ≥5 threshold remained more frequent in SjD dry eye across both age groups (<60 years = 88.9% vs. 35.0%, ≥60 years = 81.8% vs. 51.9%).

### Association Between Age and Ocular Surface Parameters

Associations between age and tear production and ocular surface staining parameters by SjD status are presented in Table 3. In linear regression models, Schirmer test values demonstrated a significant interaction between age and SjD status (interaction *P* = 0.005), indicating that the relationship between age and tear production differed between SjD dry eye and non-SjD dry eye groups. Specifically, in the non-SjD dry eye group, Schirmer values decreased with increasing age (β = −0.23 per year, *P* = 0.001), whereas no significant association between age and Schirmer values was observed in the SjD dry eye group (β = +0.27, *P* = 0.214). In contrast, across both SICCA and NEI grading systems, staining parameters showed no consistent association with age within either group, and no evidence of interaction between age and disease status.

**Table 3.** Association Between Age and Tear Production and Ocular Surface Parameters by Sjögren's Status in a Clinic-Based Sample of Patients With Sjögren's Dry Eye Versus Non-Sjögren's Dry Eye

Outcome	β Age, Non-SjD Dry Eye	*P* Value	β Age, SjD Dry Eye	*P* Value	Age × SjD Status Interaction *P* Value
Schirmer, mm/5 min	**−0.23**	**0.001**	+0.27	0.214	**0.005**
Corneal Staining Score
SICCA	0.009	0.586	−0.09	0.084	0.074
NEI	0.04	0.062	+0.004	0.954	0.576
Conjunctival Staining Score
SICCA	0.003	0.365	+0.007	0.513	0.728
NEI	−0.005	0.885	−0.153	0.183	0.220

Univariable model where β coefficients represent the change in each outcome per 1-year increase in age. Group-specific estimates were derived from linear regression models including an age and Sjögren status interaction term. The interaction *P* value tests whether the association between age and each outcome differs between SjD dry eye and non-SjD dry eye.

### Diagnostic Performance of Tear Production and Ocular Surface Disease Measures

Diagnostic performance analyses for SjD dry eye are presented in Tables 4a, 4b. Among participants with dry eye disease only (see Table 4a), Schirmer testing demonstrated weaker overall discrimination (AUROC = 0.602) compared with ocular surface staining parameters. SICCA corneal staining demonstrated the highest discriminatory ability (AUROC = 0.803), followed by SICCA combined corneal and conjunctival staining (AUROC = 0.796), NEI conjunctival staining (AUROC = 0.780), and NEI combined corneal and conjunctival staining (AUROC = 0.778).

**Table 4a.** Diagnostic Performance of Tear Production and Ocular Surface Parameters to Identify Underlying Sjögren's Among Participants in a Clinic-Based Sample of Patients With Sjögren's Dry Eye Versus Non-Sjögren's Dry Eye

Test	<60 Y AUC (95% CI)	≥60 Y AUC (95% CI)	Total Patients AUC (95% CI)
Schirmer, mm/5 min	0.772 (0.570–0.975)	0.453 (0.239–0.666)	0.602 (0.445–0.759)
SICCA staining score			
Corneal	0.872 (0.687–1.000)	0.754 (0.598–0.910)	0.803 (0.686–0.919)
Conjunctival	0.622 (0.443–0.802)	0.658 (0.487–0.830)	0.641 (0.520–0.763)
Combined corneal + conjunctival	0.861 (0.638–1.000)	0.751 (0.625–0.927)	0.796 (0.684–0.918)
NEI staining score			
Corneal	0.719 (0.504–0.935)	0.655 (0.447–0.863)	0.674 (0.528–0.821)
Conjunctival	0.875 (0.689–1.000)	0.695 (0.524–0.866)	0.780 (0.658–0.903)
Combined corneal + conjunctival	0.856 (0.672–1.000)	0.741 (0.581–0.901)	0.778 (0.658–0.898)

**Table 4b.** Diagnostic Performance of Tear Production and Ocular Surface Parameters According to Age to Identify Underlying Sjögren's Among Participants in a Clinic-Based Sample of Patients With Dry Eye Versus Controls

Test	<60 y AUC (95% CI)	≥60 y AUC (95% CI)	Total Patients AUC (95% CI)
Schirmer, mm/5 min	0.844 (0.700–0.988)	0.480 (0.271–0.689)	0.661 (0.520–0.802)
SICCA staining score			
Corneal	0.914 (0.778–1.000)	0.799 (0.668–0.930)	0.857 (0.767–0.948)
Conjunctival	0.644 (0.476–0.813)	0.676 (0.511–0.840)	0.664 (0.548–0.779)
Combined corneal + conjunctival	0.908 (0.700–1.000)	0.797 (0.715–0.950)	0.853 (0.766–0.953)
NEI staining score			
Corneal	0.824 (0.669–0.978)	0.727 (0.552–0.903)	0.774 (0.662–0.887)
Conjunctival	0.906 (0.754–1.000)	0.763 (0.620–0.905)	0.843 (0.746–0.939)
Combined corneal + conjunctival	0.896 (0.744–1.000)	0.806 (0.680–0.931)	0.849 (0.757–0.940)

AUC; area under the receiver operating characteristic curve. NEI, National Eye Institute; SICCA, Sjögren's International Collaborative Clinical Alliance.

Higher AUC values indicate better discriminatory ability of SjD dry eye (0.50 = no discrimination; 0.70–0.79 = acceptable; 0.80–0.89 = good; and ≥0.90 = excellent).

Table 4a compares SjD Dry Eye versus non-SjD Dry Eye participants only.

Table 4b compares SjD Dry Eye versus the entire cohort (non-SjD Dry Eye plus controls).

Age groups were defined as <60 y and ≥60 y.

Among participants <60 years of age, discriminatory performance was stronger across parameters, including Schirmer testing (AUROC = 0.772), SICCA corneal staining (AUROC = 0.872), SICCA conjunctival staining (AUROC = 0.622), SICCA combined corneal and conjunctival staining (AUROC = 0.861), NEI conjunctival staining (AUROC = 0.875), and NEI combined corneal and conjunctival staining (AUROC = 0.856). In participants ≥60 years of age, the performance of Schirmer testing was substantially attenuated (AUROC = 0.453), whereas staining-based measures remained more stable, particularly SICCA corneal staining (AUROC = 0.754), SICCA combined corneal and conjunctival staining (AUROC = 0.751), and NEI combined corneal and conjunctival staining (AUROC = 0.741).

When analyses were expanded to the entire cohort, including controls (see Table 4b), ocular surface staining measures again demonstrated the strongest performance. Schirmer testing showed modest overall discrimination (AUROC = 0.661), with higher performance in participants <60 years of age (AUROC = 0.844) but reduced performance in those ≥60 years of age (AUROC = 0.480). SICCA corneal staining (AUROC = 0.857), SICCA combined corneal and conjunctival staining (AUROC = 0.853), and NEI combined corneal and conjunctival staining (AUROC = 0.849) showed the highest overall discrimination.

### Independent Associations of Ocular Surface and Tear Film Parameters With SjD Dry Eye

Associations between tear production and ocular surface staining parameters and SjD dry eye are presented in Table 5. In univariable analyses, Schirmer test values were not associated with SjD dry eye (odds ratio [OR] = 0.96, 95% confidence interval [CI] = 0.89–1.05, *P* = 0.379). In contrast, ocular surface staining parameters were significantly associated with SjD dry eye, including SICCA corneal staining (OR = 2.33, 95% CI = 1.44–3.78, *P* = 0.001), NEI corneal staining (OR = 1.27, 95% CI = 1.05–1.55, *P* = 0.016), SICCA total conjunctival staining (OR = 1.81, 95% CI = 1.26–2.58, *P* = 0.001), and NEI total conjunctival staining (OR = 1.23, 95% CI = 1.09–1.40, *P* = 0.001).

**Table 5.** Association of Tear Production and Ocular Surface Parameters With Underlying Sjogren's in a Clinic-Based Sample of Patients With Dry Eye

Ocular Test	Unadjusted OR (95% CI)	*P* Value	Adjusted OR (95% CI)	*P* Value
Schirmer, mm/5 min	0.96 (0.89–1.05)	0.379	1.01 (0.92–1.10)	0.888
Corneal staining score				
SICCA	2.33 (1.44–3.78)	**0.001**	1.83 (1.01–3.31)	**0.046**
NEI	1.27 (1.05–1.55)	**0.016**	1.04 (0.81–1.33)	0.775
Conjunctival staining score				
SICCA	1.81 (1.26–2.58)	**0.001**	1.48 (0.96–2.28)	0.074
NEI	1.23 (1.09–1.40)	**0.001**	1.23 (1.05–1.42)	**0.008**

Odds ratios (ORs) were estimated using logistic regression models with SjD Dry Eye as the dependent variable.

Univariable models included each ocular parameter individually.

Multivariable models were constructed separately for NEI and SICCA grading systems and adjusted for age (continuous), Schirmer test values, and corneal and total conjunctival staining parameters within each grading system.

Worst-eye values were used for all ocular surface measures.

Statistical significance was defined as *P* < 0.05. Statistically significant *P* values are in bold.

In multivariable models adjusting for age and other ocular surface parameters, Schirmer test values remained not associated with SjD dry eye (adjusted OR = 1.01, *P* = 0.888). Among staining parameters, NEI total conjunctival staining (adjusted OR = 1.23, 95% CI = 1.05–1.42, *P* = 0.008) and SICCA corneal staining (adjusted OR = 1.83, 95% CI = 1.01–3.31, *P* = 0.046), remained independently associated with SjD dry eye.

## Discussion

In this study, we evaluated the relative utility of physician measured ocular surface and tear film parameters to distinguish SjD dry eye from non-SjD dry eye. Across analyses examining age-related effects, diagnostic accuracy, and independent associations, ocular surface staining consistently demonstrated greater clinical utility than tear production. Age-stratified analyses showed that the performance of Schirmer testing varied by age, whereas staining measures remained consistent. Diagnostic performance analyses demonstrated that SICCA corneal staining had the strongest overall ability to distinguish SjD dry eye, whereas NEI conjunctival staining showed the highest performance in younger participants (age <60 years). Multivariable models further showed that both NEI conjunctival staining and SICCA corneal staining remained independently associated with SjD dry eye after adjustment for age and other parameters, whereas Schirmer test did not.

A key finding of this study is the differential effect of age on tear production compared with ocular surface staining. Schirmer values declined with age in the non-SjD dry eye group and did not distinguish SjD dry eye from non-SjD dry eye in older participants. Age-related reduction in tear production is well described and thought to result from structural and functional changes in the lacrimal gland, including acinar atrophy and altered neural regulation of tear secretion.^17^^–^^19^ Reduced Schirmer values may reflect physiologic aging rather than autoimmune-mediated lacrimal dysfunction, limiting the specificity of Schirmer testing for SjD.^17^^,^^20^ Prior studies also demonstrated variability in the diagnostic performance of Schirmer testing, with limited sensitivity and specificity for distinguishing dry eye subtypes.^21^^,^^22^ Additionally, Schirmer testing reflects tear production but does not directly assess ocular surface inflammation or epithelial integrity.^23^ Together with our results, these findings suggest that Schirmer testing may be less reliable as a discriminatory measure in clinical practice.

Our findings are particularly relevant in the context of SjD classification criteria. The 2012 American College of Rheumatology (ACR) criteria relied on a non-weighted, rule-based approach emphasizing objective data-based markers of systemic autoimmunity and ocular surface damage (ocular staining score ≥3).^24^ Notably, Schirmer testing was not included in the 2012 criteria due to its poor correlation with disease status and limited diagnostic performance.^24^ In contrast, the 2016 ACR-EULAR criteria introduced a weighted scoring system that incorporated both laboratory and clinical findings, including ocular staining score ≥5 or Schirmer test ≤5 mm/5 minutes as an objective sign of dry eye.^14^ Although these overall criteria demonstrated high sensitivity and specificity in validation cohorts, the inclusion of Schirmer was based in part due to expert consensus and its practicality in non-ophthalmology clinical settings, rather than consistent evidence demonstrating strong independent discriminatory performance.^14^ Subsequent evaluations of this updated 2016 criteria have demonstrated variability in the performance of individual components, with objective ocular surface epithelial vital dye staining measures and serologic markers showing stronger and more consistent associations with SjD compared with tear production measures.^7^^,^^25^ Within this context, our findings provide additional evidence that Schirmer testing does not independently distinguish SjD dry eye from non-SjD dry eye and therefore is not useful as a sole ocular clinical criterion. Additionally, given that confirmatory SjD evaluation may require invasive and costly testing, including serologic evaluation and minor salivary gland biopsy, identifying ocular examination findings with stronger discriminatory value may help clinicians more confidently select patients for targeted workup. This may improve early recognition of SjD while reducing unnecessary invasive testing in lower-risk patients.

Among ocular surface staining parameters, total conjunctival staining, particularly when assessed using the NEI grading system, emerged as the most consistent discriminator between SjD dry eye and non-SjD dry eye across analytic approaches. NEI-based total conjunctival staining also demonstrated strong diagnostic performance in ROC analyses and remained independently associated with SjD dry eye in multivariable models adjusting for age and other ocular surface parameters.

Ocular surface staining reflects epithelial disruption and underlying inflammatory activity, both central features of SjD.^26^ The conjunctiva appears to be preferentially involved, with lymphocytic infiltration, goblet cell loss, and mucin deficiency contributing to ocular surface changes.^27^^,^^28^ These alterations may occur earlier and more diffusely across the conjunctiva, where epithelial damage is often more pronounced than in the cornea, allowing conjunctival staining to more sensitively and consistently capture disease-related ocular surface pathology.^29^ In contrast, corneal staining may reflect more advanced or focal epithelial damage. Consistent with this, conjunctival staining measures demonstrated more consistent independent associations with SjD dry eye in multivariable models, whereas corneal staining, particularly SICCA corneal staining, showed the strongest overall discriminatory performance in diagnostic analyses. Together, these findings suggest that conjunctival and corneal staining capture complementary aspects of disease and may provide additive clinical value.

Importantly, this study found that ocular surface staining was not uniformly absent in control participants. A substantial proportion of controls demonstrated low levels of staining, with only fewer than half showing no detectable staining by SICCA criteria and an even smaller proportion by NEI grading. Mean and median staining values were low but consistently nonzero across both corneal and conjunctival measures, indicating that mild epithelial staining may be present even in healthy individuals without dry eye. Our findings are in line with the TFOS DEWS III guidance, which recognizes that mild ocular surface staining may be present even in individuals without dry eye.^30^^,^^31^

These findings extend our prior work demonstrating that clinician-measured signs outperform patient-reported symptoms in distinguishing SjD.^11^ By directly comparing individual objective measures within the same analytic framework, our study further refines this understanding by identifying which specific ocular surface parameters provide the greatest discriminatory value.

The strengths of this study include the use of a well-characterized cohort with standardized and masked clinical assessments, allowing for direct comparison across ocular surface and tear film parameters using two grading systems. The inclusion of both continuous and threshold-based measures enhances clinical interpretability, and the use of age-stratified and multivariable analyses allowed for separation of age-related and disease-related effects. Several limitations should be considered. The sample size was modest, particularly within age-stratified subgroups, which may have limited statistical power. In addition, younger participants were under-represented in this cohort, with only 20 participants younger than 45 years and 4 younger than 35 years, and no participants younger than 30 years of age. As a result, the performance of tear film and ocular surface parameters, particularly Schirmer testing, may not be generalizable to younger populations. Control participants were also significantly younger than participants with dry eye. This likely reflects the epidemiology of dry eye disease and the clinic-based recruitment strategy, as older individuals without dry eye were less commonly encountered in this ophthalmology clinic study population. Although this age difference may have influenced comparisons with the control group, the primary analyses comparing SjD and non-SjD dry eye involved groups of similar age, reducing the likelihood that age substantially affected the principal study findings. Additionally, the higher proportion of female participants in the SjD dry eye group should be considered when interpreting comparisons between the SjD and non-SjD dry eye groups. In addition, although ocular surface staining may also be influenced by age, our study may not have been sufficiently powered to detect subtle age-related differences in staining parameters. The single tertiary-center setting may affect generalizability. Although standardized protocols were used, variability in clinical measurement is possible. In addition, although standardized grading systems were used, corneal and conjunctival staining assessments remain partly subjective and may vary between observers. Furthermore, the cross-sectional design limits the ability to assess temporal relationships or disease progression.

Future studies should validate these findings in larger, multicenter cohorts including patients with varying ages and evaluate the longitudinal behavior of ocular surface parameters in patients at risk for SjD. Development and validation of integrated clinical prediction models incorporating patient history, demographic characteristics, and ocular surface parameters may improve screening efficiency and facilitate earlier identification of SjD dry eye in routine ophthalmic practice.
